# Association of bioelectrical impedance phase angle and nutritional status in patients undergoing pancreaticoduodenectomy

**DOI:** 10.3389/fnut.2025.1554535

**Published:** 2025-07-16

**Authors:** Jialing Li, Defu Hu, Yu Yan, Mengyu Yu, Hexing Hang, Yudong Qiu, Dayu Chen, Xu Fu

**Affiliations:** ^1^Department of Pancreatic and Metabolic Surgery, Nanjing Drum Tower Hospital Clinical College of Nanjing University of Chinese Medicine, Nanjing, Jiangsu, China; ^2^Department of Pancreatic and Metabolic Surgery, Nanjing Drum Tower Hospital, Affiliated Hospital of Medical School, Nanjing University, Nanjing, Jiangsu, China; ^3^Department of General Surgery, Jiangning Hospital, Affiliated of Nanjing Medical University, Nanjing, Jiangsu, China; ^4^Department of Pathology, Nanjing Drum Tower Hospital, Affiliated Hospital of Medical School, Nanjing University, Nanjing, Jiangsu, China; ^5^Department of Pharmacy, Nanjing Drum Tower Hospital, Affiliated Hospital of Medical School, Nanjing University, Nanjing, Jiangsu, China

**Keywords:** bioelectrical impedance analysis, phase angle, pancreatoduodenectomy, nutrition status, complications

## Abstract

**Background:**

Various tools for nutritional assessment are used in individuals undergoing pancreaticoduodenectomy (PD), causing varying prevalence rates of malnutrition. This may explain the causal link between nutrition status and clinical outcomes. Phase angle (PhA), a derived metric obtained from bioelectrical impedance analysis (BIA) is used to indicate the nutrition status and evaluate disease prognosis. The aims of this study is to investigate the role of PhA in assessing the nutritional status of patients undergoing PD and to propose new strategies for the perioperative nutritional management of these patients.

**Methods:**

One hundred and seventy-three consecutive who underwent PD between March 2023 and September 2024 were evaluated and analyzed retrospectively. Comprehensive nutritional screening, evaluation, and body composition measurements were conducted within the first 48 h after admission. The Spearman correlation analysis was employed to assess the relationship between PhA and nutritional status. Receiver operating characteristic curves (ROC) were generated to assess the capacity of PhA to forecast nutrition risk and determine the cutoff value. The data were categorized into two groups according to the established cutoff value, i.e., the normal PhA group and the low PhA group. We further compared the preoperative nutritional statuses and complications between the two groups.

**Results:**

This single-center retrospective study demonstrated that PhA positively correlated with body mass index **(**BMI**)**, albumin (ALB), prealbumin (PAB), body cell mass (BCM), skeletal muscle mass (SMM), fat-free mass (FFM), and skeletal muscle mass index (SMI) (*P* < 0.001). On the other hand, PhA negatively correlated with age and extracellular water/total body water (ECW/TBW) (*P* < 0.001). The group identified as at nutritional risk and classified as malnourished group had significantly lower PhA values compared to the well-nourished group (*P* < 0.001). The ROC curves revealed that the optimal cutoff point of PhA in predicting nutrition risk was 4.85° (AUC: 0.794).

**Conclusion:**

In summary, patients undergoing PD with low PhA are more likely to develop malnutrition different degrees. Therefore, PhA may serve as a potential biomarker for preoperative nutritional assessment. While PhA shows utility in nutritional evaluation, it exhibited limited clinical significance for predicting most surgical complications in our cohort.

## Introduction

Pancreaticoduodenectomy (PD) is the standard procedure for both benign or malignant illness of the ampulla of vater, it has a high morbidity and mortality rates and a postoperative complication rate of 40%−60% (1–3). Malnutrition is widely identified as a significant risk factor for overall survival and postoperative complications following PD (4). Thus, it is important to provide specific nutritional support preoperatively, which depends on accurate assessment of the patient's nutritional status before surgery.

Currently, clinical assessments of nutritional risk and status primarily rely on various scales, including the Nutritional Risk Screening (NRS-2002) (5), the Patient-Generated Subjective Global Assessment (6) (PG-SGA) and the Global Leadership Initiative on Malnutrition (7) (GLIM). The European Society of Clinical Nutrition and Metabolism (ESPEN) and the Chinese Society for Parenteral and Enteral Nutrition (CSPEN) recommended NRS-2002 for evaluating nutritional risk in hospitalized patients. PG-SGA, specifically designed for oncology patients, is considered the preferred tool for nutritional assessment in cancer care. Similarly, GLIM has been widely adopted as the latest standard for diagnosing malnutrition in clinical practice. However, these tools often involve complex procedures and subjective assessments, which can limit their precision in diagnosing nutritional status in hospitalized patients and hinder timely interventions.

In recent years, body composition measurement has been extensively popular as an objective tool for nutritional assessment. BIA (bioelectrical impedance analysis) (8) is a simple, rapid, and non-invasive technique that calculates and analyzes body composition through the bioelectrical impedance of the human body, providing a comprehensive understanding of the patient's nutritional status. PhA (phase angle) (9) is a parameter from BIA, which indicates the cellular health and integrity of the human body. Numerous studies (10–13) have investigated the relationship between PhA and nutritional status. The results have shown that low levels of PhA correlate with malnutrition in patients undergoing surgery. Nevertheless, there is no established gold standard for detecting malnutrition risk in people undergoing PD. This work aims to explore the significance of PhA in nutritional assessment follow PD to inform subsequent treatment for nutritional support.

## Methods

### Study design, patient screening, and ethics statement

Clinical data of consecutive patients who underwent PD were retrospectively enrolled between March 2023 and September 2024 in the Department of Pancreatic Surgery, Nanjing Drum Tower Hospital.

Clinical data, including demographic characteristics, pre-operative laboratory tests, body composition measurements, and post-operative complications were all obtained from patient records. This study was conducted in accordance with Ethics Committee of Drum Tower Hospital of Nanjing University Medical School (2024-938-01).

### Inclusion and exclusion criteria

Inclusion criteria: (1) patients with complete clinical data; (2) patients≥18 years (3) underwent conventional PD; (4) pathologically confirmed malignant tumor.

Exclusion criteria: (1) patients equipped with pacemakers or who had previously received implantable electronic devices; (2) missing PhA data; (3) incomplete clinical data; (4) confused, weak, and unable to cooperate.

### Nutritional assessment

Within 48 h of admission, nutritional status was assessed by a clinical pharmacist based on the NRS-2002, PG-SGA, and GLIM tools. NRS-2002 (14) is a comprehensive tool for assessing the patient's nutritional status according to three key components: (1) impaired nutritional status score of the patient (weight loss, BMI, and the general condition or food intake); (2) disease severity score (stress metabolism due to the extent of the disease); (3) age score (patients aged ≥70 years are given additional points). By integrating these three dimensions, it is possible to accurately determine whether an individual is at nutritional risk. The final score varies of NRS-2002 ranges from 0 to 7. Generally, a score of ≥3 typically denotes the presence of nutritional risk and the need for appropriate treatment for nutritional support. The PG-SGA (15, 16) is the commonly used tool for evaluating the nutritional status of patients with malignant tumors. The subjective evaluation, which is informed by patient history and physical examination is categorized into two components. The first component is self-reporting by patients which comprises aspects including weight history, dietary modifications, gastrointestinal symptoms (including nausea, vomiting, and diarrhea), as well as levels of activity and overall physical condition. The second component is carried out by healthcare professionals and involves assessing medical history, nutritional requirements, metabolic needs, and a comprehensive physical examination of the patient. PG-SGA scores of grade A are good nutrition (0–3 points); grade B scores are suspected or moderate malnutrition (4–8 points); grade C scores are severe malnutrition (≥9 points). The GLIM (17) criteria for malnutrition based on three phenotypic criteria (low BMI, involuntary weight loss, and muscle mass loss) and two etiological criteria (disease burden, reduced food intake or absorption, and inflammatory response) were categorized according to severity thresholds of malnutrition, with stage 1 representing moderate malnutrition and stage 2 representing severe malnutrition.

### Bioelectrical impedance analysis

Measurement of body composition was conducted using the InBody770, device designed by Inbody in Korea. All participants were performed by nursing staff who had undergone standardized training in operating procedures. Two specific time intervals, 10:00–11:00 and 14:00–17:00, were selected for the assessments. Each test was completed within approximately 1 min.

To ensure precision, participants were instructed to adhere to the following guidelines: measurements should refrain from eating for at least 2 h before testing, empty bladder and bowels, without wearing heavy clothing or metal accessories, and while standing barefoot on the device. Additionally, the angle between the torso and the upper limbs was maintained at 15 degrees during the assessment. Recordings included BMI (body mass index), PhA, TBW (total body water), SMM (skeletal muscle mass), FFM (fat free mass), BFM (body fat mass), SMI (skeletal muscle mass index), BCM (body cell mass) and ECW/TBW (extracellular water/total body water).

### Clinical data collection and definition of outcomes

The clinical information retrieved from medical records included demographics (age, sex), the NRS-2002 score, the PG-SGA grade and GLIM criteria, and preoperative laboratory data (ALB, PAB). We also assessed hospitalization costs, pathological diagnosis, and incidence of complications along with the duration of post-operative hospitalization.

Postoperative complications were determined based on the Clavien-Dindo classification, with severe complications defined as grade ≥ III (18). Postoperative acute pancreatitis (PPAP), post-pancreatectomy hemorrhage (PPH), delayed gastric emptying (DGE), biliary leaks (BL), chylous fistula (CL), and clinically relevant postoperative pancreatic fistula, CR-POPF (grade B/C) were diagnosed according to the International Study Group for Pancreatic Surgery (ISGPS) (19–23).

### Statistical analysis

SPSS 27.0 was used for clinical data analyses. The Kolmogorov Smirnov method was used for the normality test, χ^2^ test or Fisher exact probability method was used for comparison of count data, the independent sample *t*-test or Mann-Whitney *U* test was used for measurement data. The diagnostic accuracy of PhA for assessing nutritional status was evaluated using the ROC curve. The optimal cutoff values were defined using Youden's index. Sensitivity and specificity were weighed equally in this analysis. Correlations were calculated using Spearman's correlation analyses. A *P*-value of <0.05 was deemed to indicate statistical significance.

## Results

### Patient characteristics

In this study, 173 patients who underwent PD were included. The study cohort comprised 97 males (56.1%) and 76 females (43.9%), with 130 patients (75.1%) classified as stages I-II and 43 patients (24.9%) classified as III-IV. The average age was 66.0 ± 10.6 years, the mean BMI was 22.1 ± 2.9 kg/m^2^, the average ALB level was 37.8 ± 3.9 g/L, and the mean PAB level was 187.3 ± 57.5 mg/L. BIA results showed that the mean PhA value among patients was 4.5 ± 0.8°. Table 1 delineates the clinical and baseline characteristics of the participants involved in the study.

**Table 1:** Baseline characteristics of the study population.

**Characteristic**	**Total (*n =* 173)**
**Gender**, ***n*** **(%)**	
Male	97 (56.1)
Female	76 (43.9)
Age (years, mean, SD)	66.0 ± 10.6
BMI (kg/m^2^, mean, SD)	22.1 ± 2.9
ALB (g/L, mean, SD)	37.8 ± 3.9
PAB (mg/L, mean, SD)	187.3 ± 57.5
PhA (°, mean, SD)	4.5 ± 0.8
TBW (L, mean, SD)	32.8 ± 6.3
SMM (kg, mean, SD)	24.2 ± 4.9
FFM (kg, mean, SD)	44.8 ± 8.2
BFM (kg, mean, SD)	15.4 ± 6.3
SMI (kg, mean, SD)	6.5 ± 0.9
BCM (kg, mean, SD)	28.6 ± 5.4
ECW/TBW	0.4 ± 0.1
**Pathological staging**, ***n*** **(%)**	
I-II	130 (75.1)
III-IV	43 (24.9)
**NRS 2002**, ***n*** **(%)**	
Malnutrition risk	138 (79.8)
Without malnutrition risk	35 (20.2)
**PG-SGA**, ***n*** **(%)**	
Grade A malnutrition	35 (20.2)
Grade B-C malnutrition	138 (79.8)
**GLIM**, ***n*** **(%)**	
Well malnutrition	62 (35.8)
Moderate to severe malnutrition	111 (64.2)
**Complications**	
Yes	69 (39.9)
No	104 (60.1)
Days of Hospitalization (day, median, IQR),	20.0 (14.5, 27.5)
Cost (dollars, median, IQR)	108,941.4 (90,354.3, 127,734.6)

BMI, body mass index; ALB, albumin; PAB, prealbumin; PhA, phase angle; TBW, total body water; SMM, skeletal muscle mass; FFM, fat free mass; BFM, body fat mass; SMI, skeletal muscle mass index; BCM, body cell mass; ECW, extracellular water; NRS-2002, nutrition risk screening; PG-SGA, patient-generated subjective global assessment; GLIM, patient-generated subjective global assessment.

**Figure 1:**
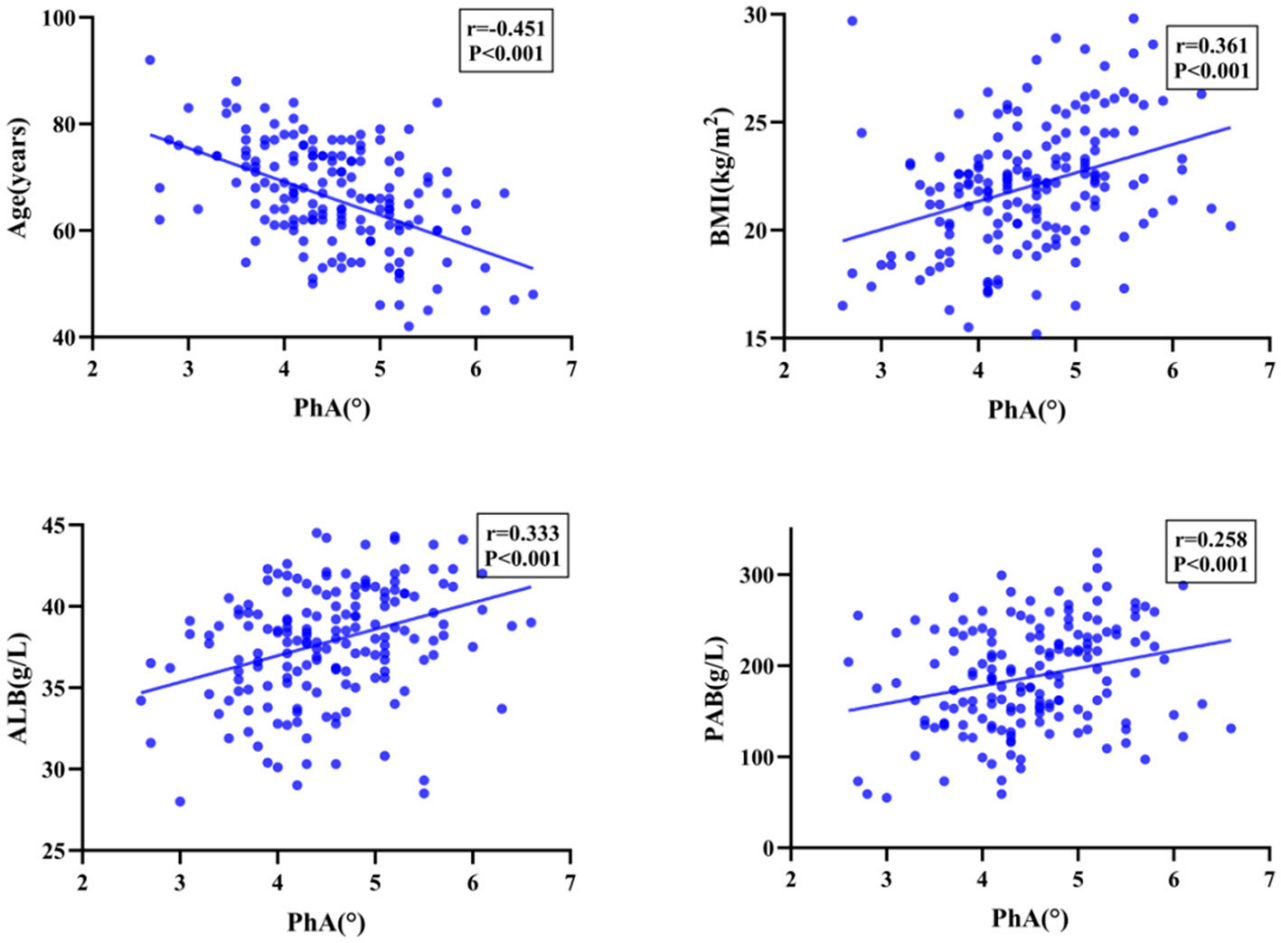
Spearman correlation coefficients between PhA and Nutrition-Related Indicators.

**Figure 2:**
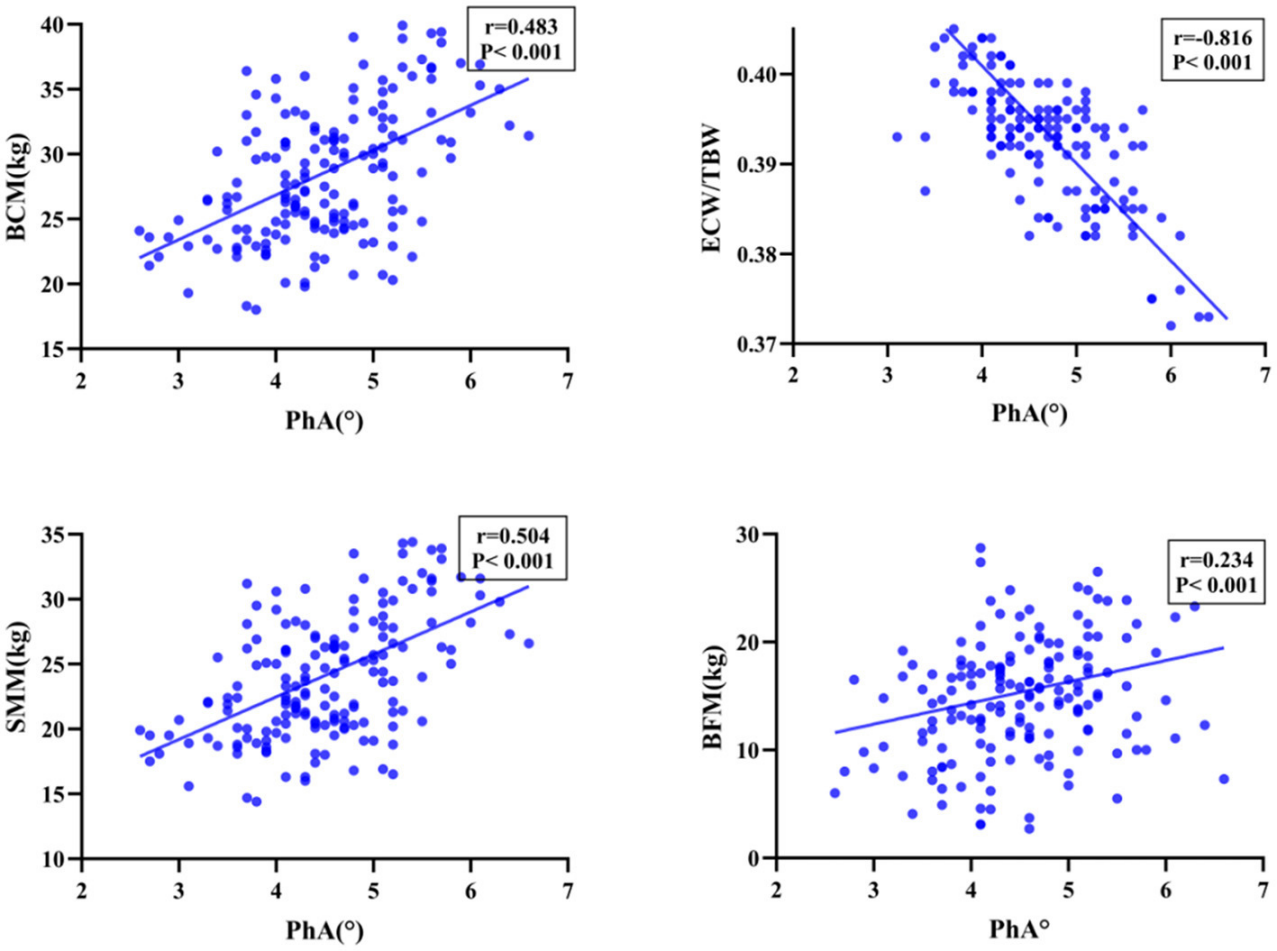
Spearman correlation coefficients between PhA and Nutrition-Related Indicators.

Among the patients who underwent PD, PhA exhibited a significant positive correlation with BMI, ALB, PAB, FFM, SMM, SMI, and BCM (*P* < 0.001), as well as demonstrated a significant negative correlation with age and ECW/TBW (*P* < 0.001). Moreover, the PhA values of patients with malnourished status were significantly lower than those of well-nourished patients, with the difference was statistically significant (*P* < 0.001, Figures 1, 2).

### The relationship between PhA and nutritional status

**Figure 3:**
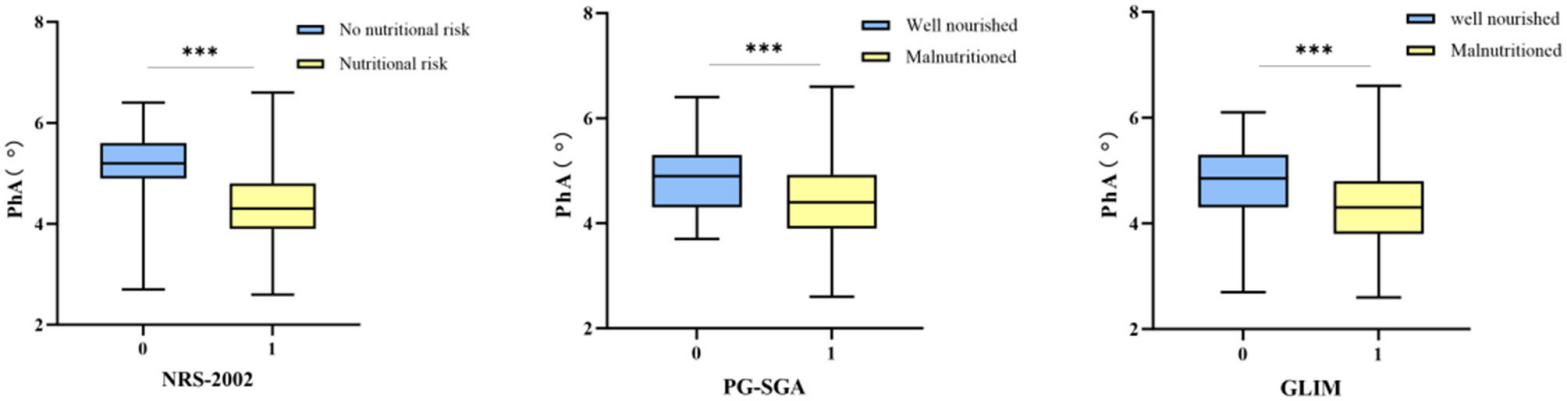
The correlation between nutritional status and PhA. Boxplots showing median and interquartile range. Significant differences are indicated with three asterisks (***) above the groups in each plot.

The nutritional assessment tool revealed diverse nutritional status of patients. Utilizing the NRS-2002 assessment tool, 138 patients (79.8%) were identified as being at risk of malnutrition. The same proportion of patients (79.8%) were classified as at risk of malnutrition based on the PG-SGA score. Moreover, 111 participants (64.2%) were diagnosed with moderate to severe malnutrition based on the GLIM criteria. Additionally, a comparison between the malnourished and well-nourished cohorts revealed a statistically significant difference in PhA values (*P* < 0.001, Figure 3).

**Figure 4:**
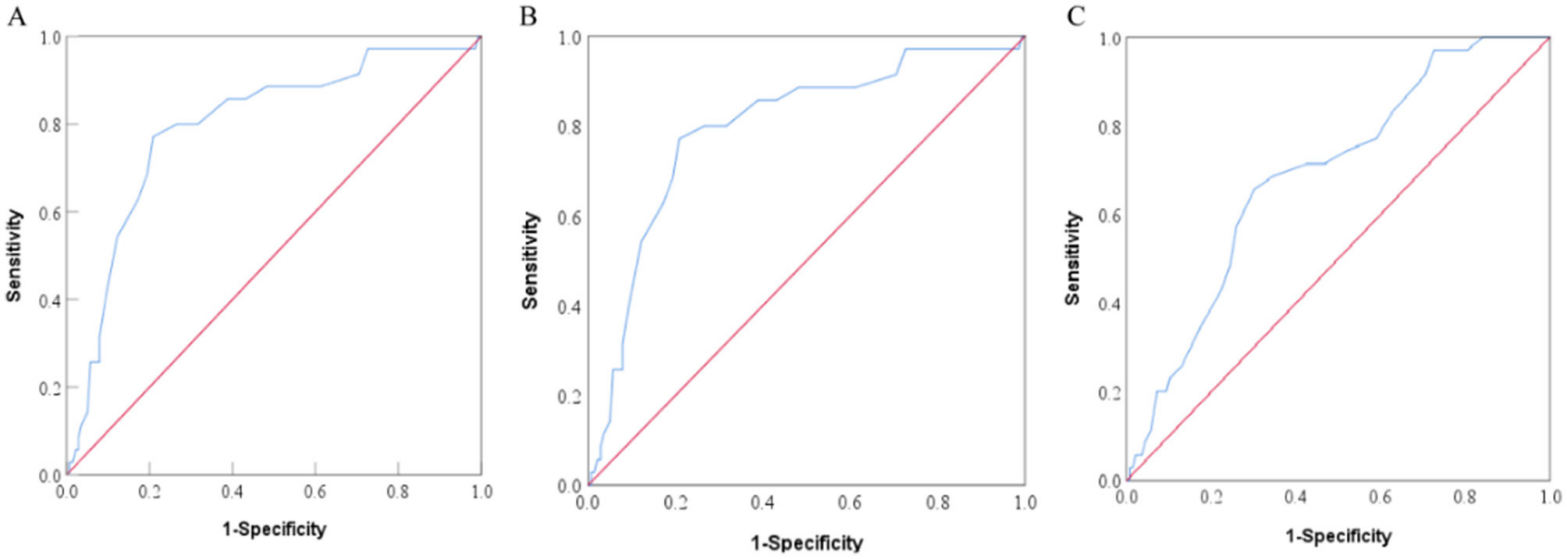
ROC curves of PhA to predict the patient nutrition status according to Nutritional Risk Screening 2002 [NRS-2002, **(A)**], Subjective Global Assessment [SGA, **(B)**], and Global Leadership Initiative on Malnutrition [GLIM, **(C)**].

ROC curve analysis was conducted based on the NRS-2002, PG-SGA, and GLIM criteria to determine the optimal cutoff value of PhA for assessing the nutritional status of patients. The AUC analysis of the PhA was 0.794 (*P* < 0.001) based on the NRS-2002, which was higher than the AUC values obtained for GLIM (0.689, *P* < 0.001) and PG-SGA (0.690, *P* < 0.001). Therefore, these results suggest that PhA is a strong predictor of malnutrition risk. The ROC curves for PhA in predicting malnutrition status are illustrated in Figure 4. The NRS-2002 nutritional screening tool identified an optimal PhA cutoff value of 4.85° for determining malnutrition risk, with a sensitivity of 77.1% and a specificity of 79.1%, as indicated by a Youden index of 0.562. A PhA measurement below 4.85° was considered low and was associated with increased risk of malnutrition.

### Nutritional status and complications in the low PhA and normal PhA groups

According to the predetermined cutoff value, patients were classified into two groups as low PhA group and normal PhA group. Table 2 outlines the prevalence of malnutrition between the two groups. The cohort with the low PhA group demonstrated significantly lower values for BMI, ALB, PAB, BCM, TBW, SMM, FFM, SMI, and BCM. In contrast, the normal PhA group exhibited significantly lower values for age and ECW/TBW.

**Table 2:** Nutritional indicators in patients with different PhA groups.

**Variables**	**Normal PhA group (*n =* 57)**	**Low PhA group (*n =* 116)**	***P*-value**	***D*-value**
**Gender**, ***n*** **(%)**				
Male	42	55	0.001	0.249
Female	15	61		
Age (years, mean, SD)	60.8 ± 9.8	68.5 ± 10.1	0	0.779
BMI (kg/m^2^, mean, SD)	23.3 ± 2.9	21.4 ± 2.8	0	0.710
ALB (g/L, mean, SD)	39.1 ± 3.6	37.2 ± 3.8	0.002	0.510
PAB (mg/L, mean, SD)	21.2 ± 56.4	176.0 ± 54.7	0	0.638
TBW (L, mean, SD)	36.2 ± 6.1	31.2 ± 5.4	0	0.874
SMM (kg, mean, SD)	27.1 ± 4.9	22.7 ± 4.2	0	0.994
FFM (kg, mean, SD)	49.2 ± 8.4	42.6 ± 7.3	0	0.862
BFM (kg, mean, SD)	17.1 ± 6.1	14.5 ± 6.3	0.01	0.419
SMI (kg, mean, SD)	7.1 ± 0.9	6.2 ± 0.8	0	0.964
BCM (kg, mean, SD)	31.7 ± 5.4	27.1 ± 4.7	0	0.935
ECW/TBW	0.4 ± 0.0	0.4 ± 0.0	0	1.442
**Pathological staging**, ***n*** **(%)**				
I-II	46	84	0.236	0.090
III-IV	11	32		
**NRS 2002**, ***n*** **(%)**				
Malnutrition risk	30	108	0	0.474
Without malnutrition risk	27	8		
**PG-SGA**, ***n*** **(%)**				
Grade A malnutrition	21	14	0	0.290
Grade B-C malnutrition	36	102		
**GLIM**, ***n*** **(%)**				
Well malnutrition	32	30	0	0.297
Moderate to severe malnutrition	25	86		
**Complications**				
Yes	26	43	0.281	0.082
No	31	73		
Days of Hospitalization (day, median, IQR)	18.0 (13.0, 30.0)	20.5 (15.0, 27.0)	0.518	0.035
Cost (dollars, median, IQR)	107,963.0 (89,403.2, 125,993.8)	110,377.9 (90,281.4, 1,265,645.4)	0.653	0.055

BMI, body mass index; ALB, albumin; PAB, prealbumin; PhA, phase angle; TBW, total body water; SMM, skeletal muscle mass; FFM, fat free mass; BFM, body fat mass; SMI, skeletal muscle mass index; BCM, body cell mass; ECW, extracellular water; NRS-2002, nutrition risk screening; PG-SGA, patient-generated subjective global assessment; GLIM, patient-generated subjective global assessment.

The overall incidence of severe postoperative complications (Clavien-Dindo classification grade ≥ 3) was 10.4%, with rates of 10.5% in the normal PhA group and 10.3% in the low PhA group (*P* = 0.971). CR-POPF occurred in 13 patients (22.8%) in the normal PhA group compared to 11 patients (9.4%) in the low PhA group (*P* = 0.017). Nonetheless, no significant differences were discovered in the other postoperative complications between the two groups (Table 3).

**Table 3:** Postoperative complication in patients with different PhA groups.

**Variables**	**Normal PhA group (*n =* 57)**	**Low PhA group (*n =* 116)**	***P*-value**	***D*-value**
Clavien-Dindo I	30	62	0.275	0.083
Clavien-Dindo II	6	12	0.919	0.008
Clavien-Dindo III-V	6	12	0.971	0.003
CR-POPF	13	11	0.017	0.181
Abdominal-infection	29	55	0.668	0.033
DGE	7	16	0.783	0.021
CL	3	18	0.052	0.148
PPH	2	8	0.369	0.068
BL	1	5	0.388	0.066
AP	2	2	0.845	0.056

CR-POPF, clinically relevant postoperative pancreatic fistula (Grade B/C); DGE, delayed gastric emptying; CL, chylous fistula; PPH, post-pancreatectomy hemorrhage; BL, biliary leakage; AP, acute pancreatitis.

The logistic regression analysis between PhA and clinical outcomes is presented in Table 4. These perioperative parameters with significant difference in the univariate analysis were evaluated in the multivariate analysis. Multivariate analysis showed no significant correlation between PhA and clinical outcomes (*P* > 0.05).

**Table 4:** Association between PhA and risk for clinical outcomes.

**Variables**	**Univariate analysis**		**Multivariate analysis**	
	**OR**	***P***	**OR**	***P***
CD-I	0.56 (0.20–1.60)	0.280	0.78 (0.26–2.33)	0.657
CD-II	0.97 (0.51–1.83)	0.919	1.39 (0.69–2.80)	0.358
CD- III-V	1.02 (0.36–2.87)	0.971		
CR-POPF	2.82 (1.17–6.78)	0.020	1.15 (0.35–3.77)	0.815
DGE	0.87 (0.34–2.26)	0.783		
BL	2.07 (0.28–15.11)	0.472		
CL	0.30 (0.08–1.07)	0.064	0.40 (0.11–1.51)	0.180
PPAP	0.39 (0.04–3.47)	0.404		
PPH	0.49 (0.10–2.39)	0.378	1.17 (0.21–6.48)	0.856
Abdominal-infection	1.15 (0.61–2.17)	0.668		

Values are hazard ratios (95% confidence intervals) unless stated otherwise. CR-POPF, clinically relevant postoperative pancreatic fistula (Grade B/C); DGE, delayed gastric emptying; BL, biliary leakage; CL, Chylous fistula; AP, acute pancreatitis; PPH, post-pancreatectomy hemorrhage.

## Discussion

This retrospective study analyzed the promising relationship between PhA and nutritional or clinical variables in patients who undergoing PD. We also investigated the relationship between PhA and post-operative complications. The findings indicated that PhA positively correlates with BMI, ALB, PAB, SMM, SMI, BCM, and negatively correlates with age and ECW/TBW. Low PhA was found to be associated with malnutrition, suggesting that it may reflect the nutritional status of candidate patients for PD, with a cutoff value of 4.85°. Furthermore, normal PhA group had a higher incidence of clinically relevant CR-POPF. While PhA shows utility in nutritional evaluation, it exhibited limited clinical significance for predicting most surgical complications in our cohort. The unexpected correlation with CR-POPF incidence, however, warrants targeted pathophysiological investigation.

PD is the definitive surgery for both benign or malignant conditions of the distal bile ducts, pancreatic head, and duodenum (1). In the present study, we found no recorded cases of postoperative mortality, indicating that PD can be safely performed at our center. Malnutrition is common among patients undergoing PD, with a reported prevalence of 80% (24). Many patients with pancreatic tumors experience considerable weight loss, attributed to tumor characteristics and associated gastrointestinal symptoms, including reduced appetite. Furthermore, the systemic inflammatory response of the body can modify metabolic states via various pathways, potentially promoting tumor progression. Malnutrition is a risk factor for infectious complications, prolonged hospitalization, and impaired quality of life (25). As a consequence, all cancer patients scheduled for pancreatic surgery should first receive early and comprehensive screening, assessment as well as intervention on their nutritional status before surgery.

Various screening tools are employed in assessing malnutrition among cancer patients. For instance, NRS-2002, PG-SGA, and GLIM are three prominent nutritional assessment tools utilized in clinical practice. A multicenter clinical research by Yu et al. (26), involving 687 cancer patients revealed that the highest prevalence of nutritional risk, both at the time of admission and discharge, was reported in individuals diagnosed with pancreatic cancer, with rates of 81.8% and 80.0%, respectively. In addition, Trestini et al. (27) revealed that the 2-year overall survival (OS) rate for patients with an NRS-2002 score of less than 3 was significantly disrupted by preoperative nutritional risk (93.7% vs. 72.7%, *P* < 0.001). Furthermore, Bicakli et al. (28) discovered that improvements in SGA were linked to reduced mortality among 304 pancreatic cancer patients; in this case, SGA acted as an independent predictor of survival in this population. Santos et al. (25) evaluated the nutritional status of 41 patients diagnosed with pancreatic cancer was assessed using the NRS-2002 and PG-SGA tools. According to the NRS-2002, we identified 82.9% of patients as being at nutritional risk; on the other hand, PG-SGA revealed that 82.9% were moderately to severely malnourished. In addition, based on the GLIM criteria, over 73.2% of patients were deemed malnourished, corroborating our findings. Nonetheless, the existing evaluation tools are based on complex and somewhat subjective scales. Consequently, it is clinically important to identify straightforward, and objective nutritional indicators with predictive benefit for nutritional assessment and body composition.

BIA utilizes the electrical properties of intra and extracellular fluids, as well as cell membranes, to assess resistance and capacitance across various electrical frequencies. This technique estimates body composition and its alterations through predictive modeling, hence a common method for analyzing body composition, assessing nutritional status, and monitoring the effects of interventions. PhA is an indicator derived from BIA, which acts as an indicator of cell membrane integrity and water distribution both intracellularly and extracellularly, thereby promoting the assessment of nutritional status. The PhA is a predictive measure of nutritional status across various diseases and its validity has been established in numerous studies. However, the critical values of PhA vary among different diseases and ethnic groups. Jiang et al. (29) highlighted the significance of PhA as an important indicator that may offer insights into both nutritional status and prognostic outcomes. Similarly, Varan et al. (30) determined that a PhA cutoff value of 4.7° indicates a malnutrition risk in hospitalized older adults. In another prospective study (31), an assessment of nutritional status in breast cancer patients undergoing their first chemotherapy demonstrated a positive correlation between PhA and the nutritional risk index (NRI), this suggests that PhA may act as a biomarker for identifying patients at risk of malnutrition. Zhou et al. (32) carried out nutritional assessments of 49 patients who underwent PD surgery and identified a cutoff value of 5.45° for PhA as a predictor of malnutrition. However, there remains a paucity of data regarding reference values and optimal cutoff points for assessing nutritional risk in PD patients. Our study presents novel findings, that the PhA in patients at risk of malnutrition is significantly lower than those without nutritional risk, thereby indicating that PhA may serve as a potential nutritional indicator for this population. Furthermore, our findings establish a critical threshold for predicting nutritional risk at a phase angle of 4.85°.

PD is a highly invasive surgery linked to a significant risk of both overall and severe postoperative complications (33). CR-POPF is one of the most prevalent and harmful conditions, with an incidence of approximately 10%−28%, causing prolonged hospital stays, delayed postoperative adjuvant therapy, and increased hospital costs (34). Several studies have identified independent risk factors including BMI (35), preoperative albumin (36), and pancreatic texture (37) for developing CR-POPF after PD. Here, we observed a higher incidence of CR-POPF in patients with normal PhA. This phenomenon may be explained by the significantly higher adiposity-related parameters (BMI, FFM, and BFM) observed in the normal PhA cohort compared to the low PhA group. However, after adjusting for potential confounding factors through multivariate regression analysis, PhA failed to demonstrate significant predictive value for postoperative complications following PD. This discrepancy with Zhou et al. (32) findings may be attributed to: (1) different PhA cutoff thresholds employed in the studies, (2) heterogeneous patient demographics, and (3) substantial inter-institutional variations in perioperative management protocols. Furthermore, although the PhA can reflect systemic cellular health status, its ability to specifically predict local complications related to PD is limited. Its predictive value may be overshadowed by more direct influencing factors such as surgical techniques, anatomical characteristics (e.g., pancreatic texture, pancreatic duct diameter), and biochemical markers (e.g., inflammatory markers). Future studies should integrate multimodal data (e.g., combining PhA, imaging parameters, and inflammatory factors) to develop more accurate predictive models and enhance clinical utility.

For patients scheduled to undergo PD with a low PhA detected preoperatively, this typically indicates impaired cell membrane integrity, inadequate nutritional reserves, and potential underlying inflammatory status. Therefore, a comprehensive nutritional intervention plan should be established for these patients prior to PD. This includes: (1) a thorough nutritional assessment integrating laboratory tests, body composition analysis, and clinical evaluation scales; (2) setting nutritional intervention targets with caloric intake of 25–30 kcal/kg/day (based on actual body weight, or adjusted weight for obese patients) and protein intake of 1.2 g/kg/day (prioritizing protein supply to correct negative nitrogen balance), while addressing dehydration and electrolyte imbalances (as low PhA is often associated with increased extracellular fluid); (3) dynamic monitoring with BIA reassessment every 2 weeks (focusing on PhA and body cell mass changes), allowing timely adjustment of the intervention plan. If persistent issues such as inadequate weight gain or low serum protein levels are observed, appropriate modifications should be made, including increasing specific nutrient intake, changing nutritional supplement types, or adjusting dietary composition, to ensure the patient achieves optimal nutritional status before surgery and improves tolerance to PD.

This study has some strengths. First, the use of BIA represents a straightforward, non-invasive, and cost-effective tool for assessing the risk of malnutrition. Second, this study indicates that PhA may have the potential to serve as a predictive indicator of malnutrition risk in patients undergoing PD, and based on the results from this cohort study, a cutoff value of 4.85° was determined. On the other hand, this work also has limitations. Initially, it is important to note that this research is a retrospective study, which may be subject to selection and information biases. Additionally, the study is conducted at a single center with a limited sample size, potentially constraining the applicability of the results to broader populations. Consequently, additional prospective studies are necessary for further validation. Notably, the potential efficacy of PhA in perioperative nutritional interventions warrants further investigations for novel clinical insights. Our findings thus indicate that PhA can predict malnutrition risk. However, the reference values used in our study may be specific to only Asian populations and may be non-generalizable to individuals from other ethnic backgrounds, considering the observed population variations in PhA. We suggest conducting the following validation work: conducting external validation studies in different ethnic populations to evaluate the applicability of the specific PhA threshold values for each population.

## Conclusion

In conclusion, our findings reveal that the PhA acts as a non-invasive, objective, and practical strategy for improving the prediction of malnutrition risk among hospitalized patients with PD. However, more studies are necessary to clarify the predictive significance of PhA in relation to clinical prognosis. This is significant for early detection of malnutrition as well as the implementation of timely nutritional interventions, which may eventually improve the nutritional status of patients and clinical outcomes.

## Data Availability

The raw data supporting the conclusions of this article will be made available by the authors, without undue reservation.
