# Supplementary material for: Association of bioelectrical impedance phase angle and nutritional status in patients undergoing pancreaticoduodenectomy
Source: Front Nutr. 2025 Jul 16;12:1554535. doi: 10.3389/fnut.2025.1554535 (PMC12307177; doi:10.3389/fnut.2025.1554535)
Supplement: Supplementary file 1 [file Table_1.docx]

Supplemental TABLE 1. Logistic regression analysis of CDI complications in patients undergoing pancreaticoduodenectomy.

| Variables | Univariate analysis | *P* | Multivariate analysis | *P* |
| --- | --- | --- | --- | --- |
|  | OR (95% Cl) |  | OR (95% Cl) |  |
| Gender | 1.07(0.44-2.64) | 0.878 |  |  |
| Age | 1.01(0.97-1.06) | 0.521 |  |  |
| BMI | 0.91(0.78-1.07) | 0.242 |  |  |
| ALB | 0.97(0.87-1.09) | 0.642 |  |  |
| PAB | 0.99(0.99-1.00) | 0.863 |  |  |
| PhA | 0.56(0.20-1.60) | 0.280 | 0.78(0.26-2.33) | 0.657 |
| SMM | 0.94(0.86-1.04) | 0.248 |  |  |
| FFM | 0.97(0.91-1.03) | 0.273 |  |  |
| BFM | 0.99(0.92-1.06) | 0.713 |  |  |
| SMI | 0.81(0.50-1.32) | 0.398 |  |  |
| BCM | 0.95(0.87-1.04) | 0.287 |  |  |
| ECW/TBW |  | 0.614 |  |  |
| Pathological staging | 1.89(0.73-4.88) | 0.186 |  |  |
| NRS-2002 | 6.10(0.79-47.03) | 0.083 |  |  |
| PG-SGA | 1.70(0.47-6.12) | 0.414 |  |  |
| GLIM | 4.06(1.15-14.30) | 0.029 | 3.79(1.04-13.83) | 0.043 |

BMI, body mass index; ALB, albumin; PAB, prealbumin; PhA, phase angle; SMM, skeletal muscle mass; FFM, fat free mass; BFM, body fat mass；SMI, skeletal muscle mass index; BCM, body cell mass; ECW, extracellular water; TBW, total body water; NRS-2002, nutrition risk screening; PG-SGA, patient-generated subjective global assessment; GLIM, patient-generated subjective global assessment.

Supplemental TABLE 1. Logistic regression analysis of CDII complications in patients undergoing pancreaticoduodenectomy.

| Variables | Univariate analysis | *P* | Multivariate analysis | *P* |
| --- | --- | --- | --- | --- |
|  | OR (95% Cl) |  | OR (95% Cl) |  |
| Gender | 0.72(0.40-1.32) | 0.295 |  |  |
| Age | 1.04(1.01-1.07) | 0.009 | 1.05(1.01-1.09) | 0.006 |
| BMI | 1.03(0.93-1.14) | 0.558 |  |  |
| ALB | 0.95(0.88-1.03) | 0.227 |  |  |
| PAB | 0.99(0.99-1.00) | 0.775 |  |  |
| PhA | 0.97(0.51-1.83) | 0.919 | 1.39(0.69-2.80) | 0.358 |
| SMM | 0.99(0.93-1.05) | 0.710 |  |  |
| FFM | 0.99(0.96-1.03) | 0.637 |  |  |
| BFM | 1.02(0.97-1.07) | 0.504 |  |  |
| SMI | 0.87(0.63-1.20) | 0.396 |  |  |
| BCM | 0.98(0.93-1.04) | 0.522 |  |  |
| ECW/TBW |  | 0.500 |  |  |
| Pathological staging | 1.02(0.51-2.03) | 0.963 |  |  |
| NRS-2002 | 1.09(0.52-2.29) | 0.816 |  |  |
| PG-SGA | 0.95(0.45-1.99) | 0.883 |  |  |
| GLIM | 1.22(0.65-2.27) | 0.531 |  |  |

BMI, body mass index; ALB, albumin; PAB, prealbumin; PhA, phase angle; SMM, skeletal muscle mass; FFM, fat free mass; BFM, body fat mass；SMI, skeletal muscle mass index; BCM, body cell mass; ECW, extracellular water; TBW, total body water; NRS-2002, nutrition risk screening; PG-SGA, patient-generated subjective global assessment; GLIM, patient-generated subjective global assessment.

Supplemental TABLE 1. Logistic regression analysis of CDIII-V complications in patients undergoing pancreaticoduodenectomy.

| Variables | Univariate analysis | *P* | Multivariate analysis | *P* |
| --- | --- | --- | --- | --- |
|  | OR (95% Cl) |  | OR (95% Cl) |  |
| Gender | 0.45(0.15-1.34) | 0.153 |  |  |
| Age | 1.02(0.97-1.07) | 0.471 |  |  |
| BMI | 0.95(0.81-1.13) | 0.595 |  |  |
| ALB | 1.04(0.91-1.18) | 0.583 |  |  |
| PAB | 1.01(0.99-1.01) | 0.214 |  |  |
| PhA | 1.02(0.36-2.87) | 0.971 |  |  |
| SMM | 1.06(0.96-1.17) | 0.252 |  |  |
| FFM | 1.03(0.97-1.08) | 0.273 |  |  |
| BFM | 0.96(0.89-1.04) | 0.357 |  |  |
| SMI | 1.26(0.74-2.13) | 0.389 |  |  |
| BCM | 1.06(0.97-1.16) | 0.213 |  |  |
| ECW/TBW | 1.09(0.95-1.23) | 0.218 |  |  |
| Pathological staging | 0.35(0.08-1.58) | 0.171 |  |  |
| NRS-2002 | 0.87(9.27-2.84) | 0.824 |  |  |
| PG-SGA | 1.30(0.35-4.77) | 0.692 |  |  |
| GLIM | 1.13(0.40-3.18) | 0.815 |  |  |

BMI, body mass index; ALB, albumin; PAB, prealbumin; PhA, phase angle; SMM, skeletal muscle mass; FFM, fat free mass; BFM, body fat mass；SMI, skeletal muscle mass index; BCM, body cell mass; ECW, extracellular water; TBW, total body water; NRS-2002, nutrition risk screening; PG-SGA, patient-generated subjective global assessment; GLIM, patient-generated subjective global assessment.

Supplemental TABLE 1. Logistic regression analysis of abdominal infection complications in patients undergoing pancreaticoduodenectomy.

| Variables | Univariate analysis | *P* | Multivariate analysis | *P* |
| --- | --- | --- | --- | --- |
|  | OR (95% Cl) |  | OR (95% Cl) |  |
| Gender | 0.76(0.42-1.39) | 0.374 |  |  |
| Age | 1.02(0.99-1.05) | 0.117 |  |  |
| BMI | 1.10(0.99-1.22) | 0.079 |  |  |
| ALB | 0.96(0.89-1.04) | 0.298 |  |  |
| PAB | 1.00(0.99-1.00) | 0.929 |  |  |
| PhA | 1.15(0.61-2.17) | 0.668 |  |  |
| SMM | 0.99(0.94-1.06) | 0.881 |  |  |
| FFM | 0.99(0.96-1.03) | 0.846 |  |  |
| BFM | 1.04(0.99-1.09) | 0.135 |  |  |
| SMI | 0.94(0.68-1.29) | 0.700 |  |  |
| BCM | 0.99(0.93-1.04) | 0.665 |  |  |
| ECW/TBW |  | 0.633 |  |  |
| Pathological staging | 1.01(0.51-2.02) | 0.966 |  |  |
| NRS-2002 | 0.87(0.41-1.82) | 0.703 |  |  |
| PG-SGA | 0.87(0.41-1.82) | 0.703 |  |  |
| GLIM | 0.91(0.49-1.70) | 0.914 |  |  |

BMI, body mass index; ALB, albumin; PAB, prealbumin; PhA, phase angle; SMM, skeletal muscle mass; FFM, fat free mass; BFM, body fat mass；SMI, skeletal muscle mass index; BCM, body cell mass; ECW, extracellular water; TBW, total body water; NRS-2002, nutrition risk screening; PG-SGA, patient-generated subjective global assessment; GLIM, patient-generated subjective global assessment.

Supplemental TABLE 1. Logistic regression analysis of CR-POPF complications in patients undergoing pancreaticoduodenectomy.

| Variables | Univariate analysis | *P* | Multivariate analysis | *P* |
| --- | --- | --- | --- | --- |
|  | OR (95% Cl) |  | OR (95% Cl) |  |
| Gender | 0.29(0.10-0.82) | 0.020 | 0.16(0.03-1.00) | 0.031 |
| Age | 1.00(0.97-1.05) | 0.665 |  |  |
| BMI | 1.20(1.04-1.40) | 0.016 | 1.20(0.95-1.53) | 0.128 |
| ALB | 1.05(0.93-1.17) | 0.445 |  |  |
| PAB | 1.00(0.99-1.01) | 0.088 |  |  |
| PhA | 2.82(1.17-6.78) | 0.020 | 1.15(0.35-3.77) | 0.815 |
| SMM | 1.14(1.04-1.25) | 0.004 | 0.98(0.28-3.40) | 0.971 |
| FFM | 1.08(1.02-1.14) | 0.005 | 0.88(0.47-1.64) | 0.688 |
| BFM | 1.06(0.99-1.13) | 0.076 |  |  |
| SMI | 2.20(1.32-3.66) | 0.002 | 1.02(0.16-6.36) | 0.981 |
| BCM | 1.13(1.04-1.23) | 0.003 | 1.19(0.59-2.41) | 0.626 |
| ECW/TBW |  | 0.444 |  |  |
| Pathological staging | 0.77(0.27-2.20) | 0.624 |  |  |
| NRS-2002 | 0.56(0.21-1.49) | 0.245 |  |  |
| PG-SGA | 0.44(0.17-1.14) | 0.091 |  |  |
| GLIM | 0.34(0.14-0.82) | 0.016 | 0.37(0.12-1.11) | 0.075 |

BMI, body mass index; ALB, albumin; PAB, prealbumin; PhA, phase angle; SMM, skeletal muscle mass; FFM, fat free mass; BFM, body fat mass；SMI, skeletal muscle mass index; BCM, body cell mass; ECW, extracellular water; TBW, total body water; NRS-2002, nutrition risk screening; PG-SGA, patient-generated subjective global assessment; GLIM, patient-generated subjective global assessment.

Supplemental TABLE 1. Logistic regression analysis of DGE complications in patients undergoing pancreaticoduodenectomy.

| Variables | Univariate analysis | *P* | Multivariate analysis | *P* |
| --- | --- | --- | --- | --- |
|  | OR (95% Cl) |  | OR (95% Cl) |  |
| Gender | 1.47(0.61-3.53) | 0.394 |  |  |
| Age | 1.02(0.97-1.06) | 0.412 |  |  |
| BMI | 0.98(0.84-1.13) | 0.751 |  |  |
| ALB | 1.02(0.91-1.14) | 0.732 |  |  |
| PAB | 1.00(0.99-1.01) | 0.496 |  |  |
| TBW | 0.99(0.92-1.06) | 0.705 |  |  |
| PhA | 0.87(0.34-2.26) | 0.783 |  |  |
| SMM | 0.98(0.89-1.07) | 0.665 |  |  |
| FFM | 0.99(0.94-1.04) | 0.679 |  |  |
| BFM | 1.01(0.94-1.08) | 0.798 |  |  |
| SMI | 0.84(0.52-1.35) | 0.473 |  |  |
| BCM | 0.98(0.91-1.07) | 0.706 |  |  |
| ECW/TBW |  | 0.941 |  |  |
| Pathological staging | 0.59(0.19-1.97) | 0.378 |  |  |
| NRS-2002 | 2.96(0.66-13.29) | 0.156 |  |  |
| PG-SGA | 1.81(0.50-6.47) | 0.363 |  |  |
| GLIM | 2.21(0.78-6.27) | 0.137 |  |  |

BMI, body mass index; ALB, albumin; PAB, prealbumin; PhA, phase angle; SMM, skeletal muscle mass; FFM, fat free mass; BFM, body fat mass；SMI, skeletal muscle mass index; BCM, body cell mass; ECW, extracellular water; TBW, total body water; NRS-2002, nutrition risk screening; PG-SGA, patient-generated subjective global assessment; GLIM, patient-generated subjective global assessment.

| Variables | Univariate analysis | *P* | Multivariate analysis | *P* |
| --- | --- | --- | --- | --- |
|  | OR (95% Cl) |  | OR (95% Cl) |  |
| Gender | 0.47(0.17-1.27) | 0.137 |  |  |
| Age | 1.01(0.97-1.06) | 0.542 |  |  |
| BMI | 0.88(0.75-1.04) | 0.144 |  |  |
| ALB | 0.97(0.86-1.09) | 0.598 |  |  |
| PAB | 0.99(0.99-1.01) | 0.777 |  |  |
| TBW | 1.01(0.93-1.08) | 0.876 |  |  |
| PhA | 0.30(0.08-1.07) | 0.064 | 0.40(0.11-1.51) | 0.180 |
| SMM | 1.00(0.91-1.01) | 0.967 |  |  |
| FFM | 1.00(0.95-1.06) | 0.943 |  |  |
| BFM | 0.97(0.89-1.04) | 0.358 |  |  |
| SMI | 0.97(0.60-1.58) | 0.912 |  |  |
| BCM | 1.01(0.92-1.09) | 0.889 |  |  |
| ECW/TBW |  | 0.184 |  |  |
| Pathological staging | 3.28(1.28-8.38) | 0.013 | 2.74(1.05-7.19) | 0.040 |
| NRS-2002 | 5.76(0.75-44.51) | 0.093 |  |  |
| PG-SGA | 2.63(0.58-11.89) | 0.208 |  |  |
| GLIM | 3.81(1.07-13.49) | 0.038 | 2.61(0.70-9.69) | 0.152 |

Supplemental TABLE 1. Logistic regression analysis of CL complications in patients undergoing pancreaticoduodenectomy.

Supplemental TABLE 1. Logistic regression analysis of PPH complications in patients undergoing pancreaticoduodenectomy.

| Variables | Univariate analysis | *P* | Multivariate analysis | *P* |
| --- | --- | --- | --- | --- |
|  | OR (95% Cl) |  | OR (95% Cl) |  |
| Gender | 0.13(0.02-1.05) | 0.056 |  |  |
| Age | 1.13(1.04-1.23) | 0.004 | 1.13(1.04-1.24) | 0.005 |
| BMI | 0.79(0.63-1.01) | 0.066 |  |  |
| ALB | 0.89(0.76-1.04) | 0.138 |  |  |
| PAB | 0.99(0.98-1.00) | 0.163 |  |  |
| TBW | 1.03(0.93-1.14) | 0.559 |  |  |
| PhA | 0.49(0.10-2.39) | 0.378 | 1.17(0.21-6.48) | 0.856 |
| SMM | 1.03(0.91-1.17) | 0.628 |  |  |
| FFM | 1.02(0.94-1.10) | 0.609 |  |  |
| BFM | 0.90(0.80-1.02) | 0.091 |  |  |
| SMI | 1.16(0.58-2.29) | 0.678 |  |  |
| BCM | 1.03(0.92-1.16) | 0.582 |  |  |
| ECW/TBW |  | 0.942 |  |  |
| Pathological staging | 0.32(0.04-2.60) | 0.287 |  |  |
| NRS-2002 | 2.37(0.29-19.38) | 0.420 |  |  |
| PG-SGA | 2.37(0.29-19.38) | 0.420 |  |  |
| GLIM | 2.33(0.48-11.33) | 0.295 |  |  |

BMI, body mass index; ALB, albumin; PAB, prealbumin; PhA, phase angle; SMM, skeletal muscle mass; FFM, fat free mass; BFM, body fat mass；SMI, skeletal muscle mass index; BCM, body cell mass; ECW, extracellular water; TBW, total body water; NRS-2002, nutrition risk screening; PG-SGA, patient-generated subjective global assessment; GLIM, patient-generated subjective global assessment.

*\*

Supplemental TABLE 1. Logistic regression analysis of BL complications in patients undergoing pancreaticoduodenectomy.

| Variables | Univariate analysis | *P* | Multivariate analysis | *P* |
| --- | --- | --- | --- | --- |
|  | OR (95% Cl) |  | OR (95% Cl) |  |
| Gender | 1.29(0.25-6.57) | 0.761 |  |  |
| Age | 1.07(0.98-1.17) | 0.143 |  |  |
| BMI | 1.09(0.83-1.44) | 0.532 |  |  |
| ALB | 1.06(0.85-1.31) | 0.619 |  |  |
| PAB | 1.00(0.99-1.02) | 0.866 |  |  |
| TBW | 0.94(0.82-1.09) | 0.423 |  |  |
| PhA | 0.39(0.04-3.47) | 0.404 |  |  |
| SMM | 0.93(0.78-1.12) | 0.453 |  |  |
| FFM | 0.96(0.86-1.07) | 0.420 |  |  |
| BFM | 1.07(0.96-1.20) | 0.201 |  |  |
| SMI | 0.65(0.26-1.57) | 0.335 |  |  |
| BCM | 0.94(0.80-1.12) | 0.486 |  |  |
| ECW/TBW |  | 0.239 |  |  |
| Pathological staging | 0.59(0.07-5.24) | 0.640 |  |  |
| NRS-2002 |  | 0.998 |  |  |
| PG-SGA |  | 0.998 |  |  |
| GLIM |  | 0.997 |  |  |

BMI, body mass index; ALB, albumin; PAB, prealbumin; PhA, phase angle; SMM, skeletal muscle mass; FFM, fat free mass; BFM, body fat mass；SMI, skeletal muscle mass index; BCM, body cell mass; ECW, extracellular water; TBW, total body water; NRS-2002, nutrition risk screening; PG-SGA, patient-generated subjective global assessment; GLIM, patient-generated subjective global assessment.

Supplemental TABLE 1. Logistic regression analysis of PPAP complications in patients undergoing pancreaticoduodenectomy.

| Variables | Univariate analysis | *P* | Multivariate analysis | *P* |
| --- | --- | --- | --- | --- |
|  | OR (95% Cl) |  | OR (95% Cl) |  |
| Gender | 0.42(0.04-4.09) | 0.454 |  |  |
| Age | 0.98(0.90-1.10) | 0.672 |  |  |
| BMI | 0.91(0.64-1.30) | 0.618 |  |  |
| ALB | 0.88(0.69-1.12) | 0.300 |  |  |
| PAB | 1.00(0.98-1.02) | 0.989 |  |  |
| TBW | 1.15(0.97-1.35) | 0.103 |  |  |
| PhA | 2.07(0.28-15.11) | 0.472 |  |  |
| SMM | 1.18(0.96-1.44) | 0.114 |  |  |
| FFM | 1.10(0.98-1.24) | 0.115 |  |  |
| BFM | 0.93(0.78-1.11) | 0.405 |  |  |
| SMI | 3.15(0.92-10.84) | 0.068 |  |  |
| BCM | 1.17(0.97-1.40) | 0.103 |  |  |
| ECW/TBW |  | 0.336 |  |  |
| Pathological staging | 1.01(0.10-9.95) | 0.995 |  |  |
| NRS-2002 | 0.24(0.03-1.79) | 0.164 |  |  |
| PG-SGA | 0.76(0.08-7.49) | 0.811 |  |  |
| GLIM | 0.18(0.02-1.76) | 0.140 |  |  |

BMI, body mass index; ALB, albumin; PAB, prealbumin; PhA, phase angle; SMM, skeletal muscle mass; FFM, fat free mass; BFM, body fat mass；SMI, skeletal muscle mass index; BCM, body cell mass; ECW, extracellular water; TBW, total body water; NRS-2002, nutrition risk screening; PG-SGA, patient-generated subjective global assessment; GLIM, patient-generated subjective global assessment.
